# The acute adverse health effects of kratom: an evaluation of case reports

**DOI:** 10.3389/fphar.2025.1620601

**Published:** 2025-08-29

**Authors:** Sarah Smallets, Sydney Litvin, Grayson Abele, Sarah Kirsh, Dennis Paustenbach

**Affiliations:** ^1^ TRC Companies, Inc., Denver, CO, United States; ^2^ TRC Companies, Inc., Jackson, WY, United States

**Keywords:** kratom (mitragyna speciosa korth), mitragynine, case report, review, adverse effect (AE), polypharmacy

## Abstract

*Mitragyna speciosa*, commonly known as kratom, has gained popularity in the United States due to its stimulant and analgesic effects. Allegations of kratom-associated adverse health effects, primarily based on case reports/series, have obtained media attention. Thus, a systematic literature search using PubMed was conducted to identify patterns among cases involving kratom use and acute adverse health effects in humans. 95 patients were identified for review. Mitragynine presence was toxicologically confirmed in 55 cases; 35 were deceased (mitragynine blood levels ranged from 3.5 to 7,500 ng/mL), and 20 were living (range of 5 to 340 ng/mL). Reported adverse effects included pulmonary, cardiovascular, brain, liver, kidney, and gastrointestinal effects, as well as seizures, loss of consciousness, lethargy, fatigue, and altered mental state. Toxicology panels revealed confounding substances that could have caused or contributed to the acute adverse effects in 32 deceased and seven surviving cases (p = 0.0002), despite attribution of many adverse effects solely to kratom. Upon analysis of the identified case reports, a pattern of weak or inadequate toxicology testing and medical history was observed. Currently, the literature provides insufficient evidence to support the claim that kratom consumption alone increases the risk of severe acute adverse health effects. More research is necessary to isolate the effects of kratom from those of polypharmacy.

## Introduction

Kratom, sourced from the leaves of *Mitragyna speciosa*, a plant endemic to Southeast Asia, has gained popularity in the U.S. over the past two decades. Before reaching the western market, kratom was traditionally used as a multi-purpose medicine, mainly to increase energy while working, and was typically consumed by chewing on the plant leaves (Swogger et al., 2022).

With the advancement of technology in the herbal and pharmaceutical spheres, kratom preparations have transitioned from the traditional means of administration (e.g., ingestion via tea); now, kratom powders, capsules, extracts, and other forms of consumption/administration are available for purchase from many different vendors across the country (Warner et al., 2016).

According to the Food and Drug Administration (FDA), “… kratom is not lawfully marketed in the U.S. as a drug product, a dietary supplement, or a food additive in conventional food,” creating a regulatory grey area surrounding the herbal product (U.S. Food and Drug Administration (FDA), 2024). Unfortunately, a lack of regulation creates quality concerns for distributors and consumers due to potential contamination by other substances and variation in alkaloid content. Adulterated kratom products may create a unique range of safety concerns, necessitating the collaboration of industry and regulatory agencies to establish a set of standards. Snow Caroti et al. (2024) published an elemental impurities assessment of kratom products, warning of exceeding regulatory permissible limits for lead, nickel, and arsenic at kratom doses of 25 g or more a day. Additionally, new products containing only 7-hydroxymitragynine, a potent metabolite of mitragynine, have entered the U.S. market, causing concern for potential unintended health risks due to the varying potency, lack of regulation, and dearth of dosing instructions (Smith et al., 2025).

1.7 million Americans, above the age of 12, were reported to have used kratom in 2021, according to the National Survey on Drug Use and Health (Substance Abuse and Mental Health Services Administration, 2022; U.S. Food and Drug Administration (FDA), 2024). Schimmel et al. (2021) estimated a past-year kratom use prevalence of 2,031,803 adults in the US population, and a lifetime prevalence of 3,353,624 adults through the Cross-sectional Survey of Non-Medical Use of Prescription Drugs (NUMRx) Program (last quarter of 2018 and first quarter of 2019). It should be noted that relying solely on survey data to estimate kratom use prevalence in the United States likely results in an underestimation of actual use patterns due to factors such as social desirability bias, recall errors, and the exclusion of high-risk or hard-to-reach populations. This is why some scholars believe that the actual prevalence of kratom use in the U.S. is much greater than the estimates stated above.

While kratom consists of over 50 alkaloids, the most abundant is mitragynine, which is hepatically metabolized to 7-hydroxymitragynine mainly by cytochrome P450 enzymes (Heywood et al., 2024). Mitragynine has demonstrated activity at serotonergic (5-HT_1a_, 5-HT_2a,_ 5-HT_2b_, and 5-HT_2c_), dopaminergic (D2 class), and adrenergic (α-1A, α-1B, α-1D) receptors (Boyer et al., 2008; Leon et al., 2021; Obeng et al., 2020; Torralva et al., 2020). Additionally, mitragynine and 7-hydroxymitragynine have binding affinity at opioid receptors (µOR, κOR, and δOR) (Chakraborty et al., 2021; Chear et al., 2021; Gharagozlou et al., 2006; Maguire et al., 1992; Obeng et al., 2020; Todd et al., 2020; Torralva et al., 2020; Volpe et al., 2011).

Self-reported reasons for kratom consumption vary, but include relief from pain, anxiety, depression, and opioid and stimulant withdrawal (Garcia-Romeu et al., 2020; Settle et al., 2024; Swogger et al., 2022). The mechanism of action that allows the primary alkaloids of kratom and their metabolites to alleviate pain and symptoms of opioid withdrawal is via interactions at opioid receptors, while reduction in anxiety and depression may be achieved through interactions with serotonergic receptors (Heywood et al., 2024; Prevete et al., 2023).

Conversely, many adverse health effects have been reported in humans using kratom, affecting multiple organ systems. For instance, the FDA and Drug Enforcement Administration (DEA) have warned consumers of the risk of liver toxicity, seizures, and death associated with kratom use (Drug Enforcement Administration (DEA), 2022; U.S. Food and Drug Administration (FDA), 2024). At the same time, the DEA has added that kratom can cause nausea, itching, sweating, dry mouth, constipation, increased urination, tachycardia, vomiting, drowsiness, loss of appetite, insomnia, and psychotic symptoms such as hallucinations, delusions, and confusion (Drug Enforcement Administration (DEA), 2022; U.S. Food and Drug Administration (FDA), 2024).

While no no-observed-adverse-effect-level (NOAEL) or lethal dose has been established for kratom use in humans, some studies have shown low doses of mitragynine (i.e., ≤53.2 mg from ≤4,000 mg dried kratom leaves per person) to be well tolerated among study subjects (Huestis et al., 2024; Prevete et al., 2024). In a single-ascending dose pilot study, no adverse effects among the study’s subjects were observed, including at the highest dose administered (24 capsules per person, which equates to 12 g of kratom), though nausea was noted at this dose (Altasciences Company Inc., 2024; Reissig et al., 2024). It has been reported that the FDA will conduct a human abuse potential study following the lack of adverse effects identified in the pilot study (American Kratom Association, 2024).

Should the trajectory of kratom popularity continue in the manner it has since its introduction to the United States, increasing the specificity in the scientific community’s understanding of how adverse effects attributed to kratom manifest itself (i.e., target organ, doses at which adverse effects are experienced in the absence of confounding substances, etc.) and what factors influence the severity and or likelihood of adverse effects is needed. It is pertinent to specify how kratom can cause acute adverse effects in both those who are kratom naïve and regular, long-term users.

Most adverse effects associated with kratom use exist in case reports/series and pharmacovigilance analyses using adverse effect reporting databases (e.g., the FDA Adverse Event Reporting System (FAERS)). Here, we present an observational study of existing case reports to assess the relationship between kratom and reported adverse effects, determine target organ systems, identify risk factor patterns, and examine confounding substances present in cases to inform potential drug-drug interactions and side effects.

## Methods

We performed a systematic literature review using PUBMED. Primary search terms were “kratom,” “mitragyna speciosa,” “mitragynine,” and “7-hydroxymitragynine,” combined with “case reports” and “case series,” using appropriate Boolean terms. In our initial search, we identified all case reports published through June 2024. No date filtration was applied to increase the likelihood of identifying an adequate number of case reports with quantified mitragynine concentrations, and, in doing so, we reviewed all available reports. A total of 137 peer-reviewed papers were pulled for further review. Publications primarily serving as review articles were considered for inclusion if they provided a case report. Additionally, studies that described withdrawal symptoms and withdrawal-related illnesses associated with kratom use were excluded, as dependency and withdrawal were endpoints outside the scope of this review.

Spontaneous reporting or pharmacovigilance systems, such as FAERS and CFSAN Adverse Event Reporting System (CAERS), were not queried for this review due to missing information and the lack of validation of information submitted. These databases are used to monitor the safety of drug and biological products, foods, and cosmetics through collecting reports of adverse events associated with a drug or product submitted by manufacturers, healthcare personnel, and consumers (Food and Drug Administration (FDA), 2024). FAERS does not collect information deemed essential for our review (i.e., blood concentrations, medical history, evidence of polysubstance use, etc.), and simply counts the number of entries that mention the searched substance and reaction. Without additional information, the exposure variable cannot be sufficiently isolated to conclude that there was an association between the exposure and the outcome of interest for any entry submitted. Additionally, according to the FAERS database disclaimer:

“Although these reports are a valuable source of information, this surveillance system has limitations, including the potential submission of incomplete, inaccurate, untimely and/or unverified information. In addition, the incidence or prevalence of an event cannot be determined from this reporting system alone due to potential under-reporting of events and lack of information about frequency of use … this website does not confirm a causal relationship between the drug product and the reported adverse event(s)” (Food and Drug Administration (FDA), 2024).

137 studies were assessed on the basis of our inclusion/exclusion criteria by two researchers, and these selections were then assessed by a third researcher, who also served as a tie-breaker should the two researchers have disagreed on the inclusion of a particular study. Studies were excluded during screening and full-text review if they did not meet the following criteria: 1. Included kratom as the exposure of interest, 2. Used a human case report or series study design, 3. Published in English, 4. Reported physical adverse event(s), 5. Reported kratom use 24 h before admission.

**FIGURE 1:**
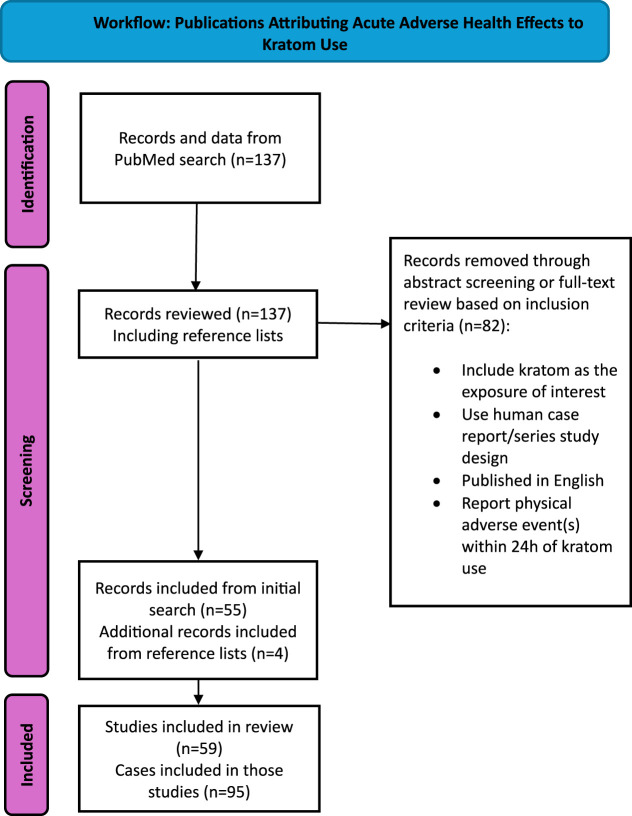
Workflow: Publications attributing acute adverse health effects to kratom use.

A total of 55 papers were identified as suitable for this review. We then evaluated the references of those chosen papers, along with relevant review papers, for other case reports and series not identified in our initial search. We identified four additional papers through this process; a total of 59 papers and 95 cases were included in this analysis (Figure 1).

We then classified the cases as either self-reported/suspected mitragynine use cases or toxicologically confirmed (e.g., qualitative and quantitative) cases in which mitragynine or 7-hydroxymitragynine concentrations were measured analytically. The two groups of cases were analyzed separately, and the results were reported independently of each other. Each study was reviewed and categorized by adverse effect-type; the categories used were death, loss of consciousness, liver effects, seizures, cardiac effects, brain effects, blood effects, kidney effects, gastrointestinal effects, pulmonary effects, urinary effects, immune effects, musculoskeletal effects, altered mental state, and miscellaneous, if the reported adverse health outcomes could not be described by any of the categories. It should be noted that altered mental states were accounted for but were not considered a physical adverse effect for the purposes of this analysis. Future research should consider the effects of kratom use on mental state.

For each case, physical attribute data (i.e., patient sex, age, BMI), kratom use data (i.e., consumption method, product type, vendor, duration and frequency of kratom dosing, as well as mitragynine and 7-hydroxymitragynine concentrations detected, if applicable), hospitalization specific data (i.e., vital signs, medical history, admission reason, secondary outcomes, clinical impression, treatment, comorbidities, and cause of death, if applicable) and other substance use specific data (i.e., concomitant substances) were collected. Total cases, percent of female cases *versus* male cases, age statistics (i.e., average, median, minimum, maximum), total deaths, consumption data (i.e., ingestion, inhalation), use parameters (i.e., multiple daily users, once daily users, less than once daily users), total cases of polysubstance use, and total cases reported for each physical adverse effect type were calculated.

Statistical analysis was limited due to a lack of available information on variables of interest (i.e., high prevalence of no reporting on product type, duration, and frequency of use), resulting in group sizes (e.g., living and deceased) that were inadequate for comparison. As such, the remaining variables with adequate participants in both survival categories were assessed by a chi-square contingency test to identify whether the difference was statistically significant, potentially indicating risk or protective factors.

## Results

The available information regarding patient kratom use, as well as the results of toxicological screening for mitragynine in both fatal and surviving cases, is summarized in Supplementary Appendix SA, B, respectively. Of 95 identified cases, 55 confirmed the presence of mitragynine via toxicological screening. 20 patients survived hospitalization, while 35 cases were fatal. Drug screening methods differed between cases; blood was tested in most reports, while urine, saliva, or other serum were screened in other reports. All mitragynine screening methods were reported by medical examiners, medical doctors, coroners, or forensic pathologists. Validation of mitragynine quantification testing was not discussed in all included case reports.

The kratom use parameters of interest were defined as product type (e.g., tea, capsules, powder, etc.), duration of kratom use (e.g., first-time user, number of years of regular use), frequency of kratom use (e.g., daily, more than once daily, less than monthly, etc.), and typical kratom dose (e.g., tablespoons of powder, number of teas, etc.).

Among 26 cases with toxicological detection of mitragynine provided with the kratom consumption method, the most reported were powder (n = 11), and drink (n = 11), followed by tea (n = 4) and leaves (n = 1). For 29 cases, the method of kratom consumption was not provided. The most reported duration of kratom use was more than 3 years (n = 7), followed by one to 3 years (n = 5). Duration of use was not provided for 38 patients. Of the cases that reported frequency of use, 11 were daily users, with two cases reporting more than one use a day. Five cases reported using kratom more than eight times per month, and one reported using kratom less than three times per month. 38 cases did not have frequency of use information. Most patients reported ingesting kratom (n = 27), one reported inhaling, and such data were not available in 27 cases. Sex, age, and kratom use parameters for the sample, stratified by survival status, are summarized in Table 1.

**TABLE 1:** Case characteristics and reported kratom use by survival status.

*Surviving*		*Deceased*	
Sex	Cases (n)	Sex	Cases (n)
Male (n, %)	19 (95%)	Male (n, %)	32 (91.4%)
Female (n, %)	1 (5%)	Female (n, %)	3 (8.6%)
Age (years)		Age (years)	
Average (SD)	30.3 (12.1)	Average (SD)	31.1 (9.8)
Range	17–64	Range	17–56
Product Type	Cases (n)	Product Type	Cases (n)
Drink	10	Did not report	26
Did not report	3	Powder	8
Powder	3	Tea	1
Tea	3		
Leaves	1		
Duration of Use	Cases (n)	Duration of Use	Cases (n)
≤1 month	3	1–12 months	1
1–12 months	2	Did not report	34
1–3 years	4		
≥3 years	7		
Did not report	4		
Frequency of Use	Cases (n)	Frequency of Use	Cases (n)
≤3 times per month	1	Daily	2
≥8 times per month	5	Did not report	33
Daily	7		
More than once a day	2		
Did not report	5		
Consumption Method	Cases (n)	Consumption Method	Cases (n)
Ingestion	18	Ingestion	9
Inhalation	1	Did not report	26
Did not report	1		

### Fatal cases

Patient death was reported in 35 cases. 32 fatal cases involved male patients (91.4%), with an average age of 31.1 years, ranging from 17 to 56 years, across all 35 cases. As stated in Table 1, most fatal cases were reported to have ingested kratom powder.

Blood mitragynine concentrations among deceased patients ranged from 3.5 ng/mL to 3,600 ng/mL in femoral blood, 190 ng/mL to 3,809 ng/mL in iliac blood, and 16 ng/mL to 1,900 ng/mL in unspecified blood. One individual had a reported concentration of 7,500 ng/mL in central blood and 3,300 ng/mL in peripheral blood. Due to the diversity of results, blood concentrations were not as informative as anticipated.

Urine mitragynine concentrations among deceased cases ranged from <10 ng/mL to 3,470 ng/mL. In three fatal cases, mitragynine was detected qualitatively (the authors reported that mitragynine was detected but did not report concentrations). Antemortem kratom dosing and use frequency were not provided for any fatal cases, except for one in which the patient reportedly ingested “one spoonful per day.” Causes of death for all fatal cases are summarized in Table 2. Urine concentrations were not considered useful except to indicate the presence of other substances.

**TABLE 2:** Reported cause of death.

Cause of death	Cases (n)	Reported in medical examiner report (n)^a^	Reported by authors (n)	Unspecified (n)^b^
Mixed drug intoxication/toxicity	15	3	12	0
Mitragynine toxicity/intoxication	6	6	0	0
Natural death (unknown)	3	3	0	0
Possible kratom or drug toxicity/not definitive	2	2		
Drug toxicity, kratom as possible contributor	2	1	1	0
Drug toxicity, kratom not contributory	2	1	0	1
Kratom intoxication with possible drug-drug interactions	1	0	0	1
Aspiration of chyme	1	1	0	0
Cardiac arrest	1	1	0	0
Asthma attack	1	1	0	0
Severe coronary atherosclerosis	1	1	0	0

^a^ Causes of death recorded in medical examiner reports were reported by either coroners, medical examiners, or forensic pathologists.

^b^ Original reporter of cause of death not specified.

Upon review of each patient’s medical history, we identified shared attributes amongst the cases including substance abuse or addiction (n = 17), alcohol abuse (n = 5) and opioid abuse (n = 4), depression and/or anxiety (n = 5), psychiatric disease (n = 3, i.e., bipolar disorder or psychosis), and seizure (n = 3). 12 case reports did not provide medical histories of the patients or reported that the medical history was not pertinent to the case. Clinical impressions, secondary outcomes, and autopsy results revealed that 26 cases experienced a pulmonary event, specifically pulmonary edema, lung congestion, and or pleural effusion.

Eight cases experienced a cardiac event (coronary atherosclerosis (n = 3); cardiomegaly (n = 2); enlarged heart (n = 1); ventricular hypertrophy (n = 1); and myocardial infarction (n = 1)). Seven experienced a brain effect, including brain/cerebral edema (n = 5), intracranial pressure (n = 1), and dysfunction (i.e., convulsions preceding death, n = 1). Four experienced a gastrointestinal event (vomit/secretions frzom mouth found at scene (n = 2); aspiration of stomach contents (n = 1); poor appetite, abdominal pain, and bloating before death (n = 1)).

Excluding kratom alkaloids and metabolites, confounding substances were detected in 32 of the fatal cases. The most frequently reported confounding substances detected in fatal cases were benzodiazepines, SSRIs, opioids, and antipsychotics. All confounding substances, grouped by therapeutic indication, are listed in Table 3.

**TABLE 3:** Confounding substances among fatal cases with confirmed mitragynine blood concentrations.

Therapeutic indication^a^	Number of cases
Benzodiazepines	20
Selective serotonin reuptake inhibitors	14
Opioids	12
Antipsychotics	10
CNS stimulants	7
Alcohol	7
Antihistamines	5
Antidepressants	5
Recreational synthetic drugs	4
Anticonvulsants	4
Sedatives	4
Cannabinoids	4
Hallucinogenic	2
Antidotes	2
Beta Blockers	2

All confounding substances were reported based on toxicology panels at the time of examination.

^a^ Reported substances were grouped by drug class and categorized by therapeutic indication.

### Surviving cases

According to the case reports, 20 patients survived hospitalization. Descriptive statistics revealed that most surviving cases were male (n = 19, 95%) and had an average sample age of 30.3 years, ranging from 17 to 64 years. As indicated in Table 1, nine of 20 (45%) of the surviving cases reported using kratom at least once a day, and most reported ingesting the product (n = 18, 90%). One case report indicated that the patient inhaled kratom. 10 of the 20 patients reported consuming a kratom “drink,” however, details regarding the contents of each drink were omitted from the case reports. Duration of use ranged from less than 1 month to longer than 3 years.

Serum mitragynine concentrations among surviving cases ranged from 5 ng/mL to 340 ng/mL, urine mitragynine concentrations ranged from 6 ng/mL to 167 ± 15 ng/mL, and one case had a saliva mitragynine concentration of 1.7 ng/ml. A urine 7-hydroxymitragynine concentration of >500 ng/mL was detected in one case. For 15 cases, kratom use was detected qualitatively.

Regarding dosing, individual patients reported an assortment of different dosing regimens, with some using 30 g of kratom a day, four teaspoons a day (5–6 g of kratom), three large teas a day, 600 mL of a homemade kratom cocktail, and one reported “increasing daily dosage.” Information regarding kratom dosing was not available for 5 cases. 10 cases reported consuming kratom in premade or homemade “drinks” which ranged from <200 mL in size to >1,000 mL. A previous study estimated the amount of mitragynine present in premade kratom drinks (250–300 mL containers) and reported a concentration of 79 mg of mitragynine in a single-dose drink (Singh et al., 2014).

An evaluation of the medical histories indicated that the most reported comorbidities were mental health issues (i.e., depression (n = 3) and anxiety (n = 1)), and drug use (i.e., alcohol abuse (n = 1) and opioid withdrawal (n = 1)). Most surviving patients presented to care facilities following seizures (n = 11) and loss of consciousness (n = 3). Comorbidities and reasons for admission found in at least one case are summarized in Tables 4, 5, respectively.

**TABLE 4:** Comorbidities among surviving cases.

Comorbidities	Count (n)
Depression	3
Alcohol abuse	1
Anxiety	1
Chronic pain	1
Insomnia	1
Long COVID	1
Opioid Withdrawal	1
Pneumonia	1
Did not report	16

Comorbidities were provided in the patients’ medical histories at the discretion of the authors of the individual case reports.

**TABLE 5:** Reason for admission among surviving cases.

Reason for admission	Count (n)
Seizures	12
Loss of consciousness	3
Vomiting/nausea	2
Drowsiness/fatigue	2
Altered mental state	1
Dark urine	1
Incomprehensible speech	1
Jaundice	1
Leg pain and edema	1
Pale stool	1
Pruritus	1

Reasons for admission to respective medical institutions were provided by the authors of each case report.

Clinical impressions showed that the most commonly reported organ systems affected were the brain (n = 13), which included symptoms such as seizures and altered mental status, and the liver (n = 5), which included elevated or otherwise abnormal liver enzyme counts, dark urine with bilirubin, cholestasis without cholecystitis, shock liver, epigastric tenderness, decreased echogenicity of the liver without cirrhosis, and intrahepatic cholestasis. Five cases reported other effects, specifically fatigue, dehydration, metabolic acidosis, and leg edema.

Confounding substances were detected in seven out of 20 surviving cases. Of note, two cases with negative toxicological screenings reported mixing kratom with diphenhydramine. Likely, the testing did not account for antihistamines and thus polysubstance use was assumed, increasing the total to nine polysubstance cases, but because this was not reported via toxicological screening, the increase was not reflected in Table 6. Opioids were the most commonly detected confounding substances (Table 6).

**TABLE 6:** Confounding substances used among surviving cases with confirmed mitragynine concentrations.

Therapeutic indication^a^	Count of therapeutic indication
Opioids	4
Cannabinoids	1
CNS stimulants	1
Antihistamine	1
Stimulant	1
Benzodiazepines	1
Serotonin-norepinephrine reuptake inhibitors	1
Antidepressants	1

All confounding substances were reported based on toxicology panels done at the time of examination.

^a^ Reported substances were grouped by drug class and categorized by therapeutic indication.

We performed a chi-squared contingency test to assess whether the deceased and surviving cases differed statistically based on polysubstance use [as a binary variable]. For the sake of being conservative, we included the two surviving cases with suspected polysubstance use, bringing the total to 9. A statistically significant difference was detected (p = 0.0002, Chi-squared statistic = 13.6). Thus, we identified polysubstance use as a variable that significantly differed between surviving and deceased cases.

Of the surviving cases with measured mitragynine concentrations and no evidence of polysubstance use, the most reported adverse effect was seizures (6/11 cases). Of these six seizure cases, no other injuries were noted, and medical histories were not provided, though the authors stated that patients with a history of epilepsy since childhood, underlying medical illness, previous brain trauma, or known structural brain diseases were excluded (Halim et al., 2021) Additionally, the authors of the case series from which all six seizure cases with no evidence of polysubstance use by toxicological screening were obtained noted that common recreational drugs were tested for but did not provide a list of tested compounds. Without further data, it cannot be ruled out that other substances were present in addition to mitragynine that were not detected because they were not included in the panel.

Loss of consciousness (n = 3), vomiting/nausea (n = 2), and drowsiness/fatigue (n = 2) were reported. The prevalence of these endpoints was too low to provide any clear indication of a relationship with kratom consumption within the present study sample. Apart from seizure activity, which occurred in 12 of the surviving cases, no health endpoint patterns were discernible among the reported mitragynine-only surviving cases.

### Comparison: Cases with and without toxicological evidence of mitragynine

The analysis of identified cases without toxicological confirmation of the presence of mitragynine is available in Supplementary Appendix SC. Among cases with and without reported mitragynine concentrations, surviving sample characteristics and kratom use parameters followed similar trends: most patients were males, the average age of patients was early to mid-30s, the most popular consumption method and product type were ingestion and powder in both groups, and the most commonly reported frequency of use was daily in both groups. Among both surviving case groups, the liver was a commonly reported organ system affected, and opioids were commonly detected as concomitant substances.

## Discussion

Kratom is a novel herbal supplement that has been the subject of a number of allegations regarding potential adverse health effects (Papsun et al., 2023). The purpose of the present study was to determine whether patterns exist among those who used kratom and who experienced an acute adverse health effect so as to better understand how kratom may cause injuries in humans. The findings of our review of the identified cases with measured mitragynine concentrations are discussed below.

### Demographics

Both surviving and deceased cases were mostly male and in their early 30s. This trend is likely representative of the demographics of the kratom-using population in the United States, rather than indicative of an underlying risk. Parent et al. (2024) analyzed data from the 2019–2020 Healthy Minds Study, which included U.S. college students, to assess demographic and behavioral factors associated with kratom use. They found that, compared to men, women had lower odds of using kratom and, compared to White individuals, Black, Asian, or Hispanic individuals also had lower odds. Garcia-Romeu et al. (2020) also conducted a survey, which found that most respondents were women, married or in a committed relationship, employed, had some college education, White, middle-aged, and middle-income. However, there is a distinction to be made between individuals who use kratom and those who experience adverse effects from kratom use. More research may be warranted as to whether men in their 30s are more likely to experience adverse effects from kratom use.

### Use parameters

Unfortunately, most fatal cases had no available kratom use parameter information, so it was not possible to compare use patterns among surviving and deceased cases to identify any differences. Regular co-ingestion of other substances with kratom may be viewed as a use parameter, though the variables were separated for our analysis.

Among the surviving cases, most reported drinking kratom and using kratom for over a year; there was no discernible pattern in frequency of use. In a 2021 online survey of participants who reported lifetime kratom use, most respondents reported currently or previously using kratom >4 times per week, consuming approximately two to three doses a day on average (Smith et al., 2022). Notably, one surviving case, with a history of illicit polysubstance abuse, reported inhaling an unspecified kratom product, while the rest of the sample reported ingesting kratom. This patient reported smoking an unknown dose of kratom, and his toxicology panel revealed serum mitragynine of 5 ng/mL, urine mitragynine of 6 ng/mL, and qualitatively positive results for caffeine and venlafaxine. At the time of this study, the effects of inhaling kratom products are unknown and not recommended.

### Fatal cases

Most fatal cases in this review (74.3%) experienced fluid accumulation within the lung tissue or the pleural space, which was not observed in any of the surviving cases, even amongst those that co-ingested kratom and other substances. According to the Royal College of Pathologists’ autopsy practice guidelines for poisonings, subacute direct toxic effects of a drug that may be fatal include pulmonary edema, hypoxic encephalopathy, and aspiration of gastric contents (The Royal College of Pathologists, 2018). Along with the high prevalence of confounding substances among the fatal cohort (91%), the pathology aligns with overdose due to polypharmacy, and the contribution of kratom, if any, is undeterminable without further data.

No evidence of polysubstance use prior to death was reported for three fatal cases. Among these cases, one patient passed due to an acute asthma attack (mitragynine blood concentration, unspecified blood: 3,600 ng/mL), one died due to an unknown cause (mitragynine blood concentration, unspecified blood: 97 ng/mL), and one death was attributed to acute mitragynine intoxication (mitragynine central blood concentration: 7,500 ng/mL, peripheral blood: 3,300 ng/mL). Information regarding the kratom product of choice, vendor, typical dose, use frequency, and use duration was not provided, so conclusions were limited. Only the acute mitragynine intoxication case had available medical history, which included prescription and illicit substance abuse, obesity, pain, one previous seizure, and several suicide attempts, including intentional overdose. Apart from the asthmatic case, the other two fatal cases experienced lung congestion; however, the asthma death and acute intoxication death had higher blood mitragynine concentrations than the case with an undetermined cause of death, demonstrating different clinical profiles across all three fatal cases with no evidence of polysubstance use.

Between July 2016 and December 2017, 27,338 unintentional and undetermined intent opioid overdose deaths were entered into the CDC’s State Unintentional Drug Overdose Reporting System (SUDORS) database, based on data from 32 states and the District of Columbia (Olsen et al., 2019). The authors reported that, “[a]lthough kratom is not an opioid, overdose deaths involving kratom (including nonopioid overdose deaths) [we]re included in SUDORS” (Olsen et al., 2019). Mitragynine was detected post-mortem in 152 decedents, and it was reported that the cause of death, determined by a medical examiner or coroner, was “kratom-involved” for 91 decedents. It was noted that for seven decedents, kratom was the only substance detected, but the authors stated, “the presence of additional substances cannot be ruled out” (Olsen et al., 2019).

In 2016, 63,632 drug overdose deaths occurred in total (Scholl et al., 2019). Thus, between 2016 and 2017, SUDORS captured seven mitragynine-only deaths, yielding a 3,905-fold difference between kratom deaths and opioid deaths, and a 9,090-fold difference between kratom deaths and total overdose deaths in the United States. Since 2019, no other kratom-associated death estimate has been published.

Using Schimmel et al. (2021)’s estimated past-year kratom use prevalence of 2,031,803 adults in the US from 2018 to 2019, and the FDA death data utilized by Henningfield et al. (2019) from the same time frame, the death rate for past-year kratom use in the United States was determined to be 0.30 per 100,000 users. Comparatively, the estimated death rate for past-year opioid use was 417 per 100,000 for *any* opioid users and 4,778 per 100,000, specifically for heroin users (Henningfield et al., 2019). Thus, the risk of death among those using any opioid or heroin is approximately 1,390 times and 15,926 times that of kratom-only associated deaths.

Readers are reminded that relying solely on survey data to estimate kratom use prevalence in the United States most likely leads to underestimation of actual use patterns, so the relative risk of death among users is almost certainly lower than that reported here.

### Surviving cases

It was reported that six polysubstance and six single substance surviving cases experienced a seizure. 11 out of 12 of these cases provided qualitative mitragynine toxicology results, so the quantitative values could not be compared. Notably, no quantitative measurements were provided for any of the reported mitragynine-only cases. Among the polysubstance users, both generalized and focal seizures were observed with about the same frequency; however, in reported mitragynine-only cases, generalized seizures were more common (5/6 cases, 83%). Focal seizures, which originate in one cerebral hemisphere, are usually associated with brain structural abnormalities, while generalized seizures, which rapidly engage neuronal networks in both hemispheres, have a wider set of etiologies, including cellular, biochemical, and structural abnormalities (R. Rao and Lowenstein, 2022).

### Confounding substances

All six seizure cases with no evidence of polypharmacy were sourced from the same publication. Halim et al. (2021) reported that “[u]rine toxicology for common recreational drugs such as morphine, cannabis, benzodiazepines, barbiturates, phencyclidine, and amphetamines were tested in all patients.” Within their case series, the authors noted that four patients reported consuming kratom and diphenhydramine syrup as a mixture, but antihistamines were not reported in urine toxicology, suggesting that those compounds were not screened for. Because the full list of detectable substances was not disclosed for this panel, it cannot be ruled out that polypharmacy was present in those cases.

There is evidence that in some deaths attributed to acute mitragynine intoxication alone, upon reexamination of blood samples, confounding substances were found. Gershman et al. (2019) reviewed Colorado death certificates from 1999 through 2017 to identify any mention of kratom. 15 kratom-related deaths were analyzed by autopsy report and toxicology testing. It was reported that 11 cases, in which no substances besides mitragynine were originally detected, tested positive for two to six additional substances, and eight blood samples tested positive for opioids. The four remaining mitragynine-only deaths were analyzed further by reviewing police investigation reports as well as comprehensive toxicology screening with high-performance liquid chromatography with tandem mass spectrometry. One case had no residual blood available for testing, so the true circumstances of death could not be determined. Among the three cases with available blood concentrations, each involved multiple substances that were previously not identified (Gershman et al., 2019). The findings of this study illustrate that there have been deaths solely attributed to kratom use, which have, upon further examination, been determined to be inappropriately ruled as such, largely due to incomplete toxicology testing.

When looking at kratom-positive deaths across the country using systems such as SUDORS, problems associated with a lack of documentation and standardization of *postmortem* toxicology testing protocols make it challenging to conclude on the contribution of kratom in serious adverse effects (Olsen et al., 2019). Olsen et al. (2019) stated that the existing *postmortem* toxicology allows researchers:

“[T]o ascertain that kratom was present primarily in deaths that occurred as a result of overdoses related to substance misuse and that kratom was most often detected in combination with multiple other substances” (Olsen et al., 2019, p. 327).

More likely than not, in fatal cases wherein kratom is detected alongside multiple other substances, death would be attributed to polypharmacy and not kratom alone. As previously stated, almost all fatal cases had at least one non-kratom substance detected, while half of the surviving cases did not. Based on basic statistical analysis, we found that the prevalence of polysubstance use differed between surviving and deceased cases (p = 0.0002). The types of confounding substances detected were different between groups as well; among polysubstance-using surviving cases, coingestion of opioids and stimulants was detected, while polysubstance-using fatal cases confirmed the presence of a wide variety of drugs, including stimulants, antipsychotics, benzodiazepines, recreational synthetic drugs, and opioids.

While these findings cannot assert that kratom use alone is safe under all circumstances, they do suggest that coadministration of other substances with kratom may increase the risk of serious adverse health outcomes, compared to kratom use alone, due to the effects of polypharmacy. The contribution of kratom ingestion to the development of adverse effects in polysubstance scenarios cannot be determined at this time. Research should be conducted to assess drug-drug interactions with kratom, especially co-administration with physician-prescribed pharmaceuticals.

### Comorbidities

Substance abuse and addiction were prevalent among the fatal cases. It follows that in most fatal cases, multiple non-kratom substances were detected. The CDC reported that in 2016, approximately 80% of synthetic opioid-involved overdose deaths involved another drug, including but not limited to alcohol, cocaine, benzodiazepines, and antidepressants, and in 2019, one-third of psychostimulant-involved deaths also involved synthetic opioids (Centers for Disease Control and Prevention (CDC), 2024; Jones et al., 2018; Kariisa et al., 2021). Crummy et al. (2020) reported that a history of polysubstance use was associated with increased risk of overdose and three times the mortality rate when compared to single-substance users. While co-administered drug combinations vary, the risk for adverse reactions due to drug-drug interactions is increased with polydrug use and should be discussed with a physician. The risk of death from polydrug use is an issue of its own and is not simply a concern of kratom use.

Among the surviving cases, mental health conditions were the most reported comorbidities, whereas the most prevalent adverse event was seizure. However, not every case that experienced a seizure reported having mental health issues. This finding may suggest that those with mental health conditions such as depression are more likely to use kratom than those without, rather than being predictive of seizure or seizure-like activity. This aligns with what is currently known regarding reasons people have reported using kratom, which includes self-treatment for anxiety, depression, and mental health problems (NIDA 2022; FDA, 2024; Rogers et al., 2021).

There is no established mechanism of action for how kratom can cause a seizure, and no evidence to assert that depression increases one’s risk of seizures. However, many substances are known to increase the risk of seizures, including antidepressants, antipsychotics, alcohol, barbiturates, benzodiazepines, amphetamines, and cocaine (Kasper et al., 2012).

### Lethality

The purpose of this paper was to evaluate the published case reports that discuss adverse effects associated with the use of kratom. As described here, at this time, it is very difficult to offer a precise value for a possible lethal dose of kratom for humans. It is also unclear whether there is a large difference in susceptibility among people (e.g., a polymorphism), which cannot be ruled out because the liver is responsible for metabolizing mitragynine to 7-hydroxymitragynine.

Clearly, there must be a dose at which kratom should have the capacity to cause death since all substances can be lethal at some dose. An exception to this general rule is the observation of a “self-limiting dose” for some substances; that is, the substance makes the user sick due to its poor taste or texture, or it may have an effect that causes the user to cease administration (i.e., headaches, nausea, vomiting).

Until recently, it was often believed within the toxicology community that kratom alone could not be taken at doses that cause death. The two papers by Papsun et al. (2023) and Papsun et al. (2019) have introduced a claim that the lethal dose may be associated with certain blood concentrations. The 2019 publication compiled case reports and mitragynine-positive blood specimens submitted to the authors’ laboratory, NMS Labs, and noted an increasing prevalence of blood mitragynine concentrations >1,000 ng/mL associated with fatal cases. Out of 6,860 cases, including *postmortem* and those tested for driving under the influence, the 2023 publication reported that 9.5% of cases had blood mitragynine levels greater than 1,000 ng/mL.

Currently, *postmortem* redistribution of kratom’s alkaloids and metabolites is not well understood. Papsun et al.‘s findings may be interpreted as hypothesis-generating, suggesting that *postmortem* redistribution among tissues, perhaps with an unknown contribution of impaired metabolism as a result of death, may play a role in increased or otherwise skewed concentrations in *postmortem* testing panels that depart from the current understanding of the pharmacokinetics of kratom in humans (as discussed in Huestis et al., 2024). There is a point to be made that only reviewing toxicology data collected *postmortem* is not adequate to comprehensively inform the safety profile of kratom. For instance, some of the fatal cases included in the current analysis may have used kratom regularly for years, but no data exists to determine how the product affected them in the years preceding death, or to determine what changed over time and why.

Papsun et al. (2023) stated that “[m]itragynine has been listed as the primary toxicological finding in several overdose deaths albeit at a lower frequency compared to overall positivity, particularly when present at elevated concentrations (>1,000 ng/mL),” which, conversely, may be interpreted by readers as an estimated lethal blood concentration. This, however, has not been substantiated. Much like the present analysis, Papsun et al. performed two observational studies, which are valuable, but there is much more to uncover regarding the blood concentrations in living users. Although some have suggested that exposure to mitragynine can cause death in humans, thus far, we are unaware of a case where mitragynine alone was responsible for a fatality that met the following criteria: 1. measured mitragynine blood concentrations, 2. complete toxicology testing with no confounding substances detected, and 3. complete medical history which would allow for understanding of preexisting conditions or comorbidities. In short, several cases of mitragynine-only fatalities have been found to have additional substances detected upon investigation (Gershman et al. 2019).

### Limitations

There were limitations to this study. First, case reports with quantified mitragynine concentrations may have been missed due to our search terms, limiting the size of our sample. Second, there was no control group to which the sample could be compared (i.e., kratom users who experienced no adverse effects). A cohort study of regular kratom users followed over time to assess use patterns, blood concentrations, and the incidence of adverse effects is needed; however, currently, there are no case reports of users who experienced no effects or positive effects. Third, case reports traditionally provide a detailed description of a diagnosis and treatment, which is especially useful for rare diseases or injuries, but, as a data source, the lack of standardization in the information obtained and reported resulted in missing data that would have been beneficial to this study and to advance the state of the science.

### Strengths

The strength of this analysis was the isolation of cases with and without toxicologically confirmed mitragynine exposure. By requiring cases analyzed to have positive qualitative or quantitative (preferred) blood or urine test data, we strengthened the reliability of our conclusions, avoided self-report bias that may have resulted in misclassification of the exposure, and prevented the inclusion of outcomes that may have been erroneously attributed to kratom products.

Additionally, this decision revealed that out of 95 cases, 40 offered no confirmation that the patient had used a kratom product (i.e., no toxicology testing reported), and yet some authors attributed the adverse effects to mitragynine.

For the purpose of furthering our understanding of the toxicology and dose-response relationship of kratom in humans, it is essential for clinicians who author case reports representing patients with alleged kratom exposure to perform a toxicology panel to confirm whether mitragynine was present or not, and to determine if other agents were present, which allows us to ascertain if contamination or co-administration played a role in the outcome. Recently, there have been calls to action to improve the quality of case reports (Shepherd et al., 2021; Thomas et al., 2022). However, among the reports included, the year of publication (i.e., 2008–2010 vs 2022–2024) appeared to have little influence on the inclusion or exclusion of the information of interest for the purposes of this analysis.

## Conclusion

There is a growing number of reports of deaths in which mitragynine was detected, such as those documented in Papsun et al. (2023) and Li et al. (2023). Accounts of kratom causing or being associated with adverse health effects have been reported. Among these case reports, most patients were found to have used kratom with other substances.

We identified a pattern of polysubstance use and confounding pharmaceutical prescriptions among the compiled case reports discussed in this paper, making the specific isolation of the effects of mitragynine alone on humans difficult, if not impossible, to determine. Based on our findings, no target organ for adverse effects could be determined in this study due to the differences in available information provided in the case reports (i.e., diagnosis, clinical impression, blood concentration, etc.). Medical history, when available, mainly aligned with reasons why the public uses kratom, rather than being predictive of the adverse event experienced, and kratom use parameters were underreported in the case reports. The main findings of this review suggested that most adverse effects coincided with polypharmacy, not kratom alone.

Further research is needed to establish the adverse effects of kratom when used alone to eliminate the uncertainty about the acute toxicity that has been presented in the literature. Additionally, it has been speculated that some publications discussed in this review provided incomplete toxicology, which would underestimate the contribution of polypharmacy and overestimate the risk of kratom to cause acute adverse effects. Incomplete toxicology in such case reports could be due to a number of factors, including patient confidentiality or lack of resources. However, details such as quantification of mitragynine levels as well as comprehensive blood screenings for other compounds are crucial in isolating the potential effects of mitragynine from other confounding factors.

In 2019, it was estimated that there were approximately two million (a highly conservative estimate) kratom users in the United States (Schimmel et al., 2021). Kratom use is expected to continue to grow in the United States, dependent upon possible future regulation. Should kratom-only exposures elicit adverse health effects, we would expect to see much higher rates of reported adverse effects from kratom use, when currently, the number of kratom-associated adverse events is low compared to recreational and illicit substances with known risks. If the estimated number of American kratom users is accurate, the number of adverse effects reported is not proportional, suggesting that there is a minimal risk of adverse effects. However, future research is needed to validate the claim that kratom alone has minimal risk for adverse effects and to establish a NOAEL. Additionally, studies are needed to assess risks associated with interactions between kratom and commonly prescribed pharmaceutical drugs.
