# Supplementary material for: The acute adverse health effects of kratom: an evaluation of case reports
Source: Front Pharmacol. 2025 Aug 29;16:1620601. doi: 10.3389/fphar.2025.1620601 (PMC12425911; doi:10.3389/fphar.2025.1620601)
Supplement: Supplementary file 1 [file Table1.docx]

Supplement A: Fatal Cases

**Table 1A: Patient Demographics, Kratom Use Patterns and Mitragynine Concentrations Detected in Fatal Cases**

| **Study** | **Patient Sex** | **Patient Age** | **Consumption Method** | **Product Type** | **Duration of Use** | **Frequency of Use** | **Mitragynine Concentration Detected** |
| --- | --- | --- | --- | --- | --- | --- | --- |
| Holler et al. (2011) | M | 20 | NA | NA | NA | NA | Iliac Blood: 390 ng/ml; urine: 1200 ng/ml |
| Neerman et al. (2013) | M | 17 | Ingestion | NA | NA | NA | Peripheral Femoral Vein Blood:600 ng/ml |
| Karinen et al. (2014) | M | NA | Ingestion | Powder | NA | NA | Blood: 1060 ng/ml; Urine:3470 ng/ml |
| McIntyre et al. (2015) | M | 24 | NA | NA | NA | NA | Peripheral Blood: 230 ng/ml; Central Blood: 190 ng/ml: Liver: 0.43 mg/kg; Vitreous: <0.050 mg/L; Urine: 370 ng/ml; Postmortem Blood: 600 ng/ml |
| Domingo et al. (2017) | M | 22 | Ingestion | Powder | NA | NA | Femoral Blood: 790 ng/ml; Urine > 400 ng/ml |
| Domingo et al. (2017) | M | 20 | NA | Powder | NA | NA | Femoral Blood: 10 ng/ml; Urine: <10 ng/ml |
| Mitchell-Mata et al. (2017) | M | 21 | NA | NA | NA | NA | Qualitatively positive |
| Hughes (2019) | M | 27 | NA | NA | NA | NA | Blood: qualitatively positive |
| Matson and Schenk (2019) | M | 33 | NA | NA | NA | NA | Blood: 1900 ng/ml |
| Walsh et al. (2019) | M | 33 | Ingestion | Powder | NA | NA | Blood (4- and 5-days post adm), serum (4 days post adm), iliac vein blood, heart blood, ocular fluid, bile, gastric fluid (all postmortem): qualitatively positive |
| Behonick et al. (2022) | M | 37 | Ingestion | Powder | NA | daily | Iliac Blood: 2325 ng/ml |
| Behonick et al. (2022) | M | 33 | Ingestion | Powder | NA | daily | Iliac Blood: 3809 ng/ml |
| Shi and Shea (2024) | M | 44 | Ingestion | Powder | 7 months | NA | Femoral Blood: 560 ng/ml |
| Mata and Chang (2023) | M | 32 | NA | NA | NA | NA | Central Blood: 7500 ng/ml; Peripheral Blood: 3300 ng/ml; Liver: 42.2 mg/kg; Gastric Contents: 33.1 mg |
| Wang and Walker (2018) | F | 56 | Ingestion | Powder | NA | NA | Femoral Blood: 2500 ng/ml |
| Kronstrand et al. (2011) | M | 22 | NA | NA | NA | NA | Blood: 70 ng/ml |
| Kronstrand et al. (2011) | M | 35 | NA | NA | NA | NA | Blood: 160 ng/ml |
| Kronstrand et al. (2011) | F | 30 | NA | NA | NA | NA | Blood: 40 ng/ml |
| Kronstrand et al. (2011) | M | 33 | NA | NA | NA | NA | Blood: 50 ng/ml |
| Kronstrand et al. (2011) | M | 27 | NA | NA | NA | NA | Blood: 180 ng/ml |
| Kronstrand et al. (2011) | M | 27 | NA | NA | NA | NA | Blood: 50 ng/ml |
| Kronstrand et al. (2011) | M | 24 | NA | NA | NA | NA | Blood: 30 ng/ml |
| Kronstrand et al. (2011) | F | 25 | Ingestion | Tea | NA | NA | Blood: 20 ng/ml |
| Kronstrand et al. (2011) | M | 32 | NA | NA | NA | NA | Blood: 50 ng/ml |
| Gershman et al. (2019) | M | NA | NA | NA | NA | NA | Whole Blood: 16 ng/ml |
| Gershman et al. (2019) | M | NA | NA | NA | NA | NA | Whole Blood: 170 ng/ml |
| Gershman et al. (2019) | M | NA | NA | NA | NA | NA | Whole Blood: 48 ng/ml |
| Jittasopa and Srisont (2021) | M | 43 | NA | NA | NA | NA | Femoral Blood: 3600 ng/ml |
| Jittasopa and Srisont (2021) | M | 48 | NA | NA | NA | NA | Femoral Blood: 15 ng/ml |
| Jittasopa and Srisont (2021) | M | 24 | NA | NA | NA | NA | Femoral Blood: 20 ng/ml |
| Jittasopa and Srisont (2021) | M | 19 | NA | NA | NA | NA | Femoral Blood: 220 ng/ml |
| Jittasopa and Srisont (2021) | M | 42 | NA | NA | NA | NA | Femoral Blood: 3.5 ng/ml |
| Jittasopa and Srisont (2021) | M | 50 | NA | NA | NA | NA | Femoral Blood: 450 ng/ml |
| Jittasopa and Srisont (2021) | M | 27 | NA | NA | NA | NA | Femoral Blood: 97 ng/ml |
| Jittasopa and Srisont (2021) | M | 37 | NA | NA | NA | NA | Femoral Blood: 150 ng/ml |

**Each row in Table 1A corresponds to the same column in descending order in Table 2A. Patient age, sex, consumption method, product type, duration of use and frequency of use were provided by the authors of each case report. Mitragynine detected concentrations were found through toxicological panels and reported by the authors of each case report.**

**Table 2A: Patient-listed Comorbidities, Medical History, Toxicology Panel, Clinical Impression, and Cause of Death in Fatal Cases**

| **Comorbidities** | **Medical History** | **Toxicology Panel Results** | **Clinical Impression** | **Cause of Death** |
| --- | --- | --- | --- | --- |
| NA | NA | Blood: Propylhexedrine (1.74 mg/L); Mitragynine (0.39 mg/L); Morphine (<0.05 mg/L); Promethazine (<0.05 mg/L); Acetaminophen (<5.0 mg/L) | At autopsy, decedent had bilateral pulmonary edema and bilateral pleural effusions. | Accidental propylhexedrine toxicity^c^ |
| Chronic pain; depression | Heroin abuse; chronic back pain; self-medication with kratom; depression; suicide attempt | Peripheral blood: Dextromethorphan (0.28mg/L); Diphenhydramine (0.33 mg/L); Temazepam (0.21 mg/L); 7-amino-clonazepam (0.21 mg/L) | A small amount of brown vomitus was noted on the decedent's face and floor. Autopsy revealed pulmonary congestion and edema and distended bladder. | Possible kratom toxicity^a^ |
| NA | Substance abuse; psychiatric disease | Blood: Zopiclone (0.043 mg/L); Citalopram (0.36 mg/L); Lamotrigine (5.4 mg/L); 7-hydroxymitragynine (0.15 mg/L) | At autopsy, decedent had patchy areas of bronchopneumonia, congested lungs, edematous, a somewhat enlarged heart with fibrotic scar in anterior wall and moderate coronary atherosclerosis. | Intoxication with kratom, possibly in combination with the other substances detected; pneumonia was precipitated by the intoxication and to have contributed to death^b^ |
| NA | Alcohol abuse; depression; suicide attempts with pills; accidental overdose | Peripheral Blood: O-Desmethylvenlafaxine (1.6 mg/L); Diphenhydramine (0.45 mg/L); Mirtazapine (0.24mg/L); Ethanol (0.02g/dL); Gastric Contents: Venlafaxine (1.1 mg/L) | At autopsy (29.5h after death declared), decedent had pulmonary edema, congestion, and moderate urinary retention. | Accidental mixed drug intoxication^b^ |
| NA | Psychosis; anxiety | Femoral Blood: Etizolam (280 µg/L); Pregabalin (3 mg/L); Pipamperone (7.4 µg/L); Lorazepam (6.9 µg/L); Triazolam (1.1 µg/L); Fluoxetine (89 µg/L); Quetiapine (18 µg/L); Olanzapine (5.8 µg/L); (Likely) 2-MMC (5.2 µg/L) | At postmortem examination, decedent had intracranial pressure and mild case of pulmonary edema. | Aspiration of chyme, possibly due to loss of consciousness^a^ |
| NA | Autism | Femoral Blood: Amphetamine (34 µg/L); MDA (40 µg/L); Methamphetamine (3300 µg/L); MDMA (1400 µg/L); Pseudoephedrine (8 µg/L); Morphine (210 µg/L); 6-MAM (41 µg/L); Paracetamol (1.9 mg/L); Codeine (24 µg/L); Caffeine (urine); GHB (480 mg/L) | NA | It was reported that there was "no clearly defined cause of death could be derived from the postmortem examination alone and a suspicion of drug intoxication was suggested by the medical examiner based on the case history."^a^ |
| NA | Drug use; treatment for drug use | Blood: Ethanol (0.047g/100mL); 3-MeO-PCP (3.2mg/L); Bupropion (1.8mg/L); Delorazepam; Paroxetine | NA | Accidental acute intoxication due to poly-drug consumption^b^ |
| NA | Asperger syndrome; bipolar disorder; substance abuse | Blood: Valproic Acid (8.8 mcg/mL); Quetiapine (12000 ng/ml) | At autopsy, decedent had dark fluid on mouth, clenched fists and intramuscular hemorrhage on cut sections of the tongue; assumed hyperthermia/fever and seizure/convulsions preceded death. | Accidental acute toxic effects of quetiapine complicated by mitragynine use^c^ |
| NA | Substance abuse (heroin, cocaine, benzos, methamphetamine, alcohol, marijuana, prescription opioids, phencyclidine (PCP), spice, ecstasy, mushrooms); depression; anxiety | Inferior Vena Cava Blood: D-9 tetrahydrocannabinol (0.0026 mg/L); Caffeine; Cotinine; Naloxone | NA | Mitragynine toxicity^a^ |
| Hypertension; obesity; anxiety disorder; suspected depression | NA | Blood, Serum, Iliac Vein Blood, Heart Blood, Ocular Fluid, Bile, Gastric: Alprazolam; Etizolam; Fentanyl - given in hospital; Labetalol; Laudanosine - given in hospital; Lorazepam - given in hospital; Metoprolol; Mirtazapine; Morphine; Nicardipine - given in hospital; Norfentanyl; Normirtazapine; Norsertraline; Ofloxacin - given in hospital; Sertraline; U-47700 (1.85 ng/mL) | Overdose | Accidental complications of multiple drug toxicity^a^ |
| NA | Remote traumatic brain injury; alcohol abuse; withdrawal seizures disorder; cigarette smoker | Iliac Blood: Cotinine | NA | Acute mitragynine intoxication^a^ |
| Anxiety; depression | Polysubstance abuse (ethanol, cocaine, kratom, marijuana) | Iliac Blood: Cotinine; Naloxone | NA | Acute mitragynine intoxication^a^ |
| NA | Metastatic seminoma treated with surgery and chemotherapy; schizoaffective disorder bipolar type | Femoral Blood: Hydromorphone (79 mg/mL); Olanzapine (240 ng/mL) | Decedent had abdominal distension, but no signs of acute liver failure, and >4L ascites with associated pleural effusion. | Hydromorphone toxicity with mitragynine use listed as a contributing factor^a^ |
| Severe depression; oppositional disorder; obesity | Alcohol abuse; illicit drug abuse; prescription opioid drug use; back pain; headaches; obesity; one seizures three to four months before admission; many suicide attempts, including intentional overdose | NA | Significant autopsy findings were cardiomegaly, hepatomegaly, congested lungs, cerebral edema, and obesity. | Acute mitragynine intoxication^a^ |
| Group B Streptococcus; staphylococcus | COPD; dyspnea; cough; prescribed Percocet and lorazepam were abused; used cannabidiol oil drops and "methadone-like" powder | Femoral Blood: Oxycodone (0.19 ± 0.01 mg/L); Lorazepam (63 ± 5 ng/L) | Decedent was found with white residue on the outside of her lips. Internal examination demonstrated cardiomegaly with biventricular hypertrophy and mild atheromatous disease of the coronary arteries with no myocardial scars or established acute myocardial infarction; luminous within the tracheobronchial tree and both lungs were heavy and appeared hyperinflated with prominent fibrosis; congestive hepatopathy from passive venous congestion, attributable to right-side heart failure; granular cortical scarring of kidneys. | Accidental death secondary to multidrug toxicity^b^ |
| NA | Drug abuse | Blood: O-DMT (0.4 ug/g); Alprazolam (ug/g); Ethanol (0.09 ug/g) | Decedent had congestion of lungs. | Accidental drug poisoning^b^ |
| NA | Drug abuse | Blood: O-DMT (0.7 ug/g); Alimemazine (0.3 ug/g); DMA (0.1 ug/g); Venlafaxine (0.7 ug/g); O-DMV (0.1 ug/g) | Decedent had edema and congestion of lungs. | Accidental drug poisoning^b^ |
| NA | Drug abuse | Blood: O-DMT0.5 ug/g; Fluoxetine (0.6 ug/g); Norfluoxetine (0.5 ug/g); Phenazone (19.8 ug/g); Olanzapine (0.2 ug/g); Diazepam (0.3 ug/g); Nordiazepam (0.3 ug/g); Pregabalin (5.0 ug/g); Amphetamine (0.04 ug/g) | Decedent had congestion of lungs and liver steatosis. | Accidental drug poisoning^b^ |
| NA | Drug abuse | Blood: O-DMT (1.5 ug/g); Alimemazine (0.2 ug/g); DMA (0.2 ug/g); Olanzapine (0.1 ug/g); Nordiazepam (0.05 ug/g); THC (0.002 ug/g) | Decedent had edema and congestion of lungs, hepatitis, liver steatosis, Mb Hodgkin and aspiration of stomach contents. | Accidental drug poisoning^b^ |
| NA | Drug abuse | O-DMT (4.3 ug/g); Alimemazine (0.2 ug/g); Mirtazapine (0.1 ug/g); Venlafaxine (0.1 ug/g); Diazepam (0.09 ug/g); Nordiazepam (0.2 ug/g), Buprenorphine (0.0004 ug/g) | Decedent had brain and lung edema. | Accidental drug poisoning^b^ |
| NA | Drug abuse | Blood: O-DMT (1.2 ug/g); Zopiclone (0.04 ug/g); Ethanol (0.01 ug/g) | Decedent had brain and lung edema. | Accidental drug poisoning^b^ |
| NA | Drug abuse | Blood: O-DMT (1.1 ug/g); Alprazolam (0.14 ug/g); Amphetamine (0.20 ug/g); THC (0.0006 ug/g) | Decedent had brain and lung edema. | Accidental drug poisoning^b^ |
| NA | NA | Blood: O-DMT (0.08 ug/g); Venlafaxine (1.0 ug/g); O-DMV (1.1 ug/g); Zopiclone (0.06 ug/g) | Decedent had congestion of lungs. | Accidental drug poisoning^b^ |
| NA | Drug abuse; alcohol abuse | Blood: O-DMT (1.1 ug/g); Citalopram (0.8 ug/g); Alprazolam (0.07 ug/g); THC (0.007 ug/g) | Decedent had brain and lung edema. | Accidental drug poisoning^b^ |
| NA | Opioid abuse; benzodiazepine abuse; seizures associated with benzodiazepine withdrawal | Whole Blood: 5-MeO-AMT; Psychedelic Tryptamine (56.19 ng/ml), Etizolam, Benzodiazepine (1.3 ng/ml); Ethanol (18 mg/dl) | NA | Cardiac arrest^a^ |
| NA | NA | Whole Blood: 1,3-dimethylamylamine, Methylhexanamine (157 ng/ml) | NA | Mitragynine toxicity listed (unknown cause)^a^ |
| NA | NA | Whole Blood: Trimethoxyamphetamine (2.83 ng/ml) | NA | Mitragynine toxicity listed (unknown cause)^a^ |
| NA | NA | NA | NA | Acute asthmatic attack^a^ |
| NA | NA | Femoral Blood: Sildenafil (0.13 mg/L) | Decedent had pulmonary edema and fatty liver. | Severe coronary atherosclerosis^a^ |
| NA | NA | Femoral Blood: Heroin and metabolites; Codeine; Methamphetamine; Morphine (0.07 mg/L) | Decedent had mild coronary atherosclerosis and severe liver congestion. | Multiple drug intoxication^a^ |
| NA | NA | Femoral Blood: Alprazolam (0.078 mg/L); Clonazepam (0.0090 mg/L); multiple Antihistamines | Decedent had left ventricular hypertrophy and pulmonary edema. | Multiple drug intoxication^a^ |
| NA | NA | Femoral Blood: Methamphetamine (0.32 mg/L); Chlorpheniramine (qualitative) | Decedent had old myocardial infarction, fatty liver, moderate coronary atherosclerosis, and pulmonary edema. | Methamphetamine intoxication^a^ |
| NA | NA | Femoral Blood: Diazepam (0.16 mg/L); Amitriptyline (0.50 mg/L) | Decedent had pulmonary edema. | Natural death (unknown cause)^a^ |
| NA | NA | NA | Decedent had pulmonary edema. | Natural death (unknown cause)^a^ |
| NA | NA | Femoral Blood: Ethanol (25.10 mg%) | NA | Natural death (unknown cause)^a^ |

**Each row in Table 2A corresponds to the same column in descending order in Table 1A. Comorbidities at the time of hospitalization/autopsy, medical history, toxicological panel results and clinical impressions were provided by the authors of each case report.**

**^a^ Cause of death reported by medical examiner, coroner or forensic pathologist.**

**^b^ Cause of death reported by authors of case report.**

**^c^ Where cause of death was reported was unspecified.**
