# Supplementary material for: The acute adverse health effects of kratom: an evaluation of case reports
Source: Front Pharmacol. 2025 Aug 29;16:1620601. doi: 10.3389/fphar.2025.1620601 (PMC12425911; doi:10.3389/fphar.2025.1620601)
Supplement: Supplementary file 2 [file Table2.docx]

Supplement B: Surviving Cases

**Table 1B: Patient Demographics, Kratom Use Patterns and Mitragynine Concentrations Detected in Surviving Cases**

| **Author** | **Consumption Method** | **Product Type** | **Dose** | **Duration of Use** | **Frequency of Use** | **Mitragynine Concentration Detected** |
| --- | --- | --- | --- | --- | --- | --- |
| Boyer et al. (2008) | Ingestion | Tea | NA | 3.5 years | > Daily | Qualitative |
| Nelsen et al. (2010) | Ingestion | Tea with *Datura stramonium* | NA | NA | NA | Urine: 167 ± 15 ng/ml |
| Overbeek et al. (2019) | Ingestion | NA | NA | NA | NA | Urine: qualitative |
| Palasamudram Shekar et al. (2019) | Ingestion | Powder | NA | 3 weeks | Daily | Urine (7-hydroxymitragynine): > 500 ng/ml |
| Muller et al. (2020) | Ingestion | Powder | 30g | 3 years | > Daily | Saliva: 1.7 ng/ml |
| Nacca et al. (2020) | Ingestion | Powder | 5.63g | 1 year | Daily | Serum: 340 ng/ml |
| Jensen et al. (2021) | Ingestion | Tea | NA | NA | NA | Qualitative (day 10 admission) |
| Patel et al. (2021) | NA | NA | NA | 1 week | NA | Urine: "high levels" |
| Tobarran et al. (2022) | Inhalation | NA | NA | NA | NA | Urine: 6 ng/mL; Serum: 5 ng/mL |
| Thewjitcharoen et al. (2022) | Ingestion | Diphenhydramine, soda, and Kratom mix | 600ml drink | 2 weeks | Daily | Qualitative |
| Halim et al. (2021) | Ingestion | Diphenhydramine and Kratom mix | 500-100 ml drink | 5 years | ≥ 8 times per month | Qualitative |
| Halim et al. (2021) | Ingestion | Drink | <200 ml drink | 3 months | ≥ 8 times per month | Qualitative |
| Halim et al. (2021) | Ingestion | Drink | >1000 ml drink | 7 years | ≥ 8 times per month | Qualitative |
| Halim et al. (2021) | Ingestion | Diphenhydramine and Kratom mix | 200-200 ml drink | 1 year | ≥ 8 times per month | Qualitative |
| Halim et al. (2021) | Ingestion | Drink | 200-200 ml drink | 5 months | ≤ 3 times per month | Qualitative |
| Halim et al. (2021) | Ingestion | Diphenhydramine and Kratom mix | >1000 ml drink | 7 years | ≥ 8 times per month | Qualitative |
| Halim et al. (2021) | Ingestion | Drink | 200-200 ml drink | 2 years | Daily | Qualitative |
| Halim et al. (2021) | Ingestion | Drink | 200-200 ml drink | 2 years | Daily | Qualitative |
| Halim et al. (2021) | Ingestion | Drink | >1000 ml drink | 5 years | Daily | Qualitative |
| Halim et al. (2021) | Ingestion | Diphenhydramine and Kratom mix | 200-200 ml drink | 2 years | Daily | Qualitative |

**Each row in Table 1B corresponds to the same column in descending order in Table 2B. Patient age, sex, consumption method, product type, duration of use, and frequency of use were provided by the authors of each case report. Mitragynine detected concentrations were found through toxicological panels and reported by the authors of each case report.**

**Table 2B: Patient-listed Comorbidities, Medical History, Toxicology Panel, Reason for Admission, Clinical Impression, and Organ Systems Affected in Surviving Cases**

| **Comorbidities** | **Medical History** | **Toxicology Panel Results** | **Reason for Admission** | **Clinical Impression** | **Organ Systems Affected** |
| --- | --- | --- | --- | --- | --- |
| Chronic pain; opioid replacement and withdrawal therapy | Chronic pain from thoracic outlet syndrome treated with Hydromorphone; subcutaneous injection of 10 mg Hydromorphone per day from crushed pills | Modafinil | GTC seizure | Following a brief post-ictal period, physical examination of patient was normal. | Brain (seizure) |
| Chronic pain; depression | Colostomy repair one month prior; chronic pain and depression managed with Amitriptyline, Oxycodone, and kratom; significant alcohol and tobacco use | Urine: Cannabinoids, Tricyclic Antidepressants, and Oxycodone | Seizure; coma | Patient had constricted and minimally reactive pupils, lower extremity spasticity with manipulation, sinus tachycardia, and a seizure. | Brain (seizure); loss of consciousness |
| Depression | Polysubstance abuse; previous hospitalization for altered mental status (positive for Bupropion, Venlafaxine, and kratom) | NA | Altered mental status; decreased respiratory rate; bradypnea | Patient had reported "opioid toxidrome." | Pulmonary; mental |
| NA | NA | NA | Found unresponsive at home | Patient had significantly elevated serum AST and ALT, anion gap, and lactic acid, creatine kinase of 700 U/L, hyperkalemia, and scored 3 on the Glasgow coma scale | Liver; cardiovascular; blood; kidney; loss of consciousness |
| Depression; anxiety | Tobacco, Marijuana, Cocaine, Benzodiazepine, Methamphetamine, and Amphetamine use | NA | Drowsiness; fatigue | NA | Miscellaneous (fatigue and drowsiness) |
| NA | Hepatitis C; alcohol and opioid use disorder | NA | Altered mental status; incomprehensible speech; vomiting | Patient had elevated Troponin T of 91 ng/L | Brain; gastrointestinal |
| NA | Opioid and alcohol use disorder; PTSD | Urine: Buprenorphine and Benzodiazepines (inpatient medications) | Drug addiction | Patient had significantly elevated liver enzymes (AST and ALT were 4 and 10 times the upper normal limit) and secondary GI outcomes from opioid withdrawal. | Liver; gastrointestinal; miscellaneous (dehydration) |
| NA | Alcohol and opioid abuse; "questionable seizures" were reported | NA | Found unresponsive at home | Patient had elevated WBC, platelets, creatinine, AST and ALT, and an assumed underlying seizure with postictal state resulting in hypoxemia and concerns for anoxic brain injury. | Liver; cardiac; kidney; pulmonary; musculoskeletal; miscellaneous (severe metabolic acidosis); loss of consciousness |
| NA | Sporadic cocaine use; ethanol use; prescription opioid and heroin use | Urine: Caffeine and Venlafaxine | Loss of consciousness for six hours; left leg pain; left leg edema | NA | Liver; musculoskeletal; miscellaneous (fasciotomy); loss of consciousness |
| Long COVID and mild COVID-19 pneumonia in 2 months prior to death; excessive drinking | 270 grams of alcohol per week on average | Urine: Diphenhydramine | Fatigue; nausea; pruritus; dark urine; jaundice; pale stool | Patient had epigastric tenderness on right upper quadrant, dark urine with urobilinogen and bilirubin, elevated transaminase and direct bilirubin and overall decreased echogenicity of the liver without cirrhosis. | Liver; gastrointestinal; urinary; miscellaneous (fatigue) |
| NA | NA | Opioids (44 ng/ml) | FBTC seizure | Patient had dizziness. | Brain (seizure) |
| NA | NA | NA | GTC seizure | NA | Brain (seizure) |
| NA | NA | NA | FBTC seizure | Patient had palpitations and dizziness. | Brain (seizure) |
| NA | NA | NA | GTC seizure | NA | Brain (seizure) |
| NA | NA | NA | GTC seizure | NA | Brain (seizure) |
| NA | NA | ATS (631 ng/ml); Opioids (87 ng/ml) | FBTC seizure | Patient had palpitations. | Brain (seizure) |
| NA | NA | NA | GTC seizure | NA | Brain (seizure) |
| NA | Methamphetamine (4 days prior to kratom ingestion) | NA | GTC seizure | NA | Brain (seizure) |
| NA | NA | NA | GTC seizure | NA | Brain (seizure) |
| NA | NA | NA | GTC seizure | NA | Brain (seizure) |

**Each row in Table 2B corresponds to the same column in descending order in Table 1B. Comorbidities at the time of hospitalization, medical history, toxicological panel results, clinical impressions, and reasons for admission were provided by the authors of each case report. Reported symptoms were grouped and categorized by organ system affected.**
