# Supplementary material for: The acute adverse health effects of kratom: an evaluation of case reports
Source: Front Pharmacol. 2025 Aug 29;16:1620601. doi: 10.3389/fphar.2025.1620601 (PMC12425911; doi:10.3389/fphar.2025.1620601)
Supplement: Supplementary file 3 [file Table3.docx]

**Case Reports without Measured Mitragynine Concentrations**

Out of 95 identified cases, 40 cases did not provide toxicological evidence of kratom use. Instead, these reports relied on patient-reported data to qualify kratom use. 39 patients survived hospitalization, while one case was fatal. The sample characteristics of the 40 case reports are summarized in Table 1C.

**Table 1C. Sample Characteristics**

| **Variable** | **Value** |
| --- | --- |
| Sex (n, %) |  |
| Male | 29 (72%) |
| Female | 11 (28%) |
| Age (years) |  |
| Average (SD) | 37.45 (14.1) |
| Range | 18-63 |

Among all 40 cases, the most reported product type was powder (n=10), followed by capsules (n=7), liquid (n=2), tea (n=2), and leaves (n=1). For 18 cases, the product type was not provided. The most reported duration of kratom use was less than or equal to one month (n=8), followed by less than or equal to three months (n=4) and more than or equal to a year (n=4). Duration of use was not provided for 19 cases. Of the cases that reported frequency of use, the majority were daily users (n=14), with 2 cases reporting more than one use a day. 22 cases did not have frequency of use information. Most cases reported ingesting kratom (n=25), one case reported inhaling kratom, and 14 cases did not report how they used kratom. Kratom use parameters for the sample are summarized in Table 2C.

**Table 2C. Kratom Use Parameters**

| **Duration of Use** | **Count (n)** |
| --- | --- |
| ≤1 month | 8 |
| ≤3 months | 4 |
| ≤6 months | 3 |
| ≥1 year | 4 |
| First use | 2 |
| Did not report | 19 |
| **Frequency of Use** | **Count (n)** |
| Daily | 14 |
| Less than once a day | 2 |
| More than once a day | 2 |
| Did not report | 22 |
| **Product Type** | **Count (n)** |
| Powder | 10 |
| Capsules | 7 |
| Leaves | 1 |
| Liquid | 2 |
| Tea | 2 |
| Did not report | 18 |
| **Consumption Method** | **Count (n)** |
| Ingestion | 25 |
| Inhalation | 1 |
| Did not report | 14 |

*Fatal Cases*

One case was fatal. A 26-year-old man was admitted to the hospital in cardiorespiratory arrest. Upon admission, arrest was managed, and after approximately one hour, spontaneous circulation was achieved. A CT scan showed imminent cerebral herniation. Chemical parameters improved with treatment for approximately an hour, and then cardiorespiratory function decreased once again, which ultimately resulted in mortality. His cause of death was reported as cardiorespiratory failure and hypoxic brain damage. It was reported by an unknown source that the patient had consumed an unknown amount of kratom in the prior 24 hours before admission. No other kratom use parameters or toxicological evidence supporting mitragynine intoxication was provided. A urine screen was positive for codeine, an opioid.

*Surviving Cases*

39 cases survived hospitalization. Descriptive statistics revealed that most surviving cases were male (n=28, 72%), with an average sample age of 37.74 years, ranging from 18 to 63 years. 41% of surviving cases reported using kratom at least once a day, and 64% reported ingesting the product. The most reported duration of kratom use was less than or equal to one month (n=8), followed by less than or equal to three months (n=4) and less than or equal to six months (n=4). Duration of use was not provided for 18 cases. Among the 31 surviving cases, the most reported product type was powder (n=10), followed by capsules (n=7), liquid (n=2), tea (n=2), and leaves (n=1). For 17 cases, the product type was not provided.

For cases where grams per day could be calculated from reported dosing information, doses ranged from 1.5 to 14 grams per day. Other cases reported 28 milliliters (3-4 8 mL bottles) per day, “several pills a day”, 12 drinks per week, one drink per day, more than 10 pills (approximately 0.5 grams per pill) per day, “higher kratom use,” and 3200 mg of capsules plus a full bottle of liquid extract once. Information regarding the kratom dose prior to the adverse event was not available for 22 cases.

Medical history review revealed that the most reported comorbidities were generally substance abuse, with six cases reporting opioid use disorder, three reporting polysubstance use, three reporting history of abuse, one reporting substance use, and one reporting alcohol abuse. Depression (n=6), chronic pain (n=6), and anxiety (n=5) were also commonly reported comorbidities identified in medical histories. Comorbidities and previous conditions with greater than one case are summarized in Table 3C.

**Table 3C. Comorbidities and Past Conditions (n>1) Among Surviving Cases**

| **Comorbidities/Medical Histories** | **Count (n)** |
| --- | --- |
| Depression | 6 |
| Opioid use disorder | 6 |
| Chronic pain | 6 |
| Anxiety | 5 |
| Hypertension | 5 |
| Did not report | 4 |
| History of substance abuse | 4 |
| Hyperlipidemia | 3 |
| Polysubstance use | 3 |
| ADHD | 3 |
| Epilepsy | 2 |
| Asthma | 2 |

Comorbidities were provided in the patients’ medical histories dictated by the authors of the individual case reports.

Medical history review revealed that the most reported reasons for admission were nausea and vomiting (n=14 of 91 reasons listed), loss of consciousness (n=8), seizures (n=5), and fatigue (n=5). The reasons for admission with greater than one case among surviving cases are summarized in Table 4C.

**Table 4C. Reason for Admission (n>1) Among Surviving Cases**

| **Reason for Admission** | **Count (n)** |  |
| --- | --- | --- |
| Nausea/vomiting | 14 |  |
| Loss of consciousness | 8 |  |
| Seizures | 5 |  |
| Fatigue | 5 |  |
| Abdominal cramping | 4 |  |
| Dark urine | 4 |  |
| Fever | 4 |  |
| Jaundice | 4 |  |
| Reduced appetite | 3 |  |
| Diarrhea | 3 |  |
| Weakness | 3 |  |
| Altered mental status | | 3 |
| Cardiac arrest | 2 |  |
| Epigastric pain | 2 |  |
| Constipation | 2 |  |

Reasons for admission to respective medical institutions were provided by the authors of each case report.

Clinical impressions showed that the most reported organ system affected was the liver (n=16), which included cholestatic hepatitis, acute cholestatic liver injury, acute hepatitis/drug-induced hepatitis, acute liver failure, jaundice, elevated/abnormal liver enzyme levels, and drug-induced liver injury (DILI); followed by the gastrointestinal system (n=10), which included symptoms such as reduced appetite, vomiting/nausea, abdominal pain, epigastric pain, dark urine, diarrhea, and constipation. Six of the nine cases in which gastrointestinal symptoms occurred showed signs of liver injury. Miscellaneous events, specifically weakness and dizziness, gallstones, pancreas injury, fasciotomy, dilation of the common bile duct, dental abscesses, fatigue, dry eyes, and dry mouth, also affected 7 cases.

Confounding substances were detected in 13 out of 39 surviving cases. Cannabinoids (n=6 of all confounding substances detected) and Opioids (n=5) were the two most detected confounding substances in cases where mitragynine concentrations were not confirmed using toxicological evidence [Table 5C].

**Table 5C.** **Confounding Substances Among Surviving Cases without Confirmed Mitragynine Concentrations**

| **Therapeutic Indications** | **Count of Therapeutic Indication** |
| --- | --- |
| Cannabinoids | 6 |
| Opioids | 5 |
| Benzodiazepines | 3 |
| Stimulants | 2 |
| Alcohol | 2 |
| Steroids | 2 |
| CNS stimulants | 1 |
| Antihistamine | 1 |
| Anticonvulsants | 1 |

All confounding substances were reported based on toxicology panels performed at the time of examination.

^a^ Reported substances were grouped by drug class and categorized by therapeutic indication.

**References**

Abdullah HMA, Haq I, Lamfers R. 2019. Cardiac arrest in a young healthy male patient secondary to kratom ingestion: is this 'legal high' substance more dangerous than initially thought? BMJ Case Reports. 12: 1-4.

Afzal H, Esang M, Rahman S. 2020. A Case of Kratom-induced Seizures. Cureus. 12(1): 1-9.

Aggarwal G, Robertson E, McKinlay J, Walter E. 2018. Death from Kratom toxicity and the possible role of intralipid. Journal of the Intensive Care Society. 19(1): 61-63.

Ahmed S, Tran QV, McLean M. 2023. The Great Imitator: A Case of Accidental Kratom Overdose. Cureus. 15(8): 1-5.

Botejue M, Walia G, Shahin O, Sharma J, Zackria R. 2021. Kratom-Induced Liver Injury: A Case Series and Clinical Implications. Cureus. 13(4): 1-3.

Burke DJ, Mahonski SG, Van Cott AC. 2021. Breakthrough Seizure Associated With Kratom Use in Patients With Epilepsy. Neurology: Clinical Practice. 11(1): 78-84.

Castillo A, Payne JD, Nugent K. 2017. Posterior reversible leukoencephalopathy syndrome after kratom ingestion. Baylor University Medical Center Proceedings. 30(3): 355-357.

Dasgupta A, Ye Z. 2024. Severe jaundice with life-threatening liver failure after Kratom use: Reversed by plasma exchange. Transfusion and Apheresis Science. 63(3): 1-3.

Dorman C, Wong M, Khan A. 2015. Cholestatic hepatitis from prolonged kratom use: a case report. Hepatology. 61(3): 1086-1087.

Eudaley ST, Brooks SP, Hamilton LA. 2023. Case Report: Possible Serotonin Syndrome in a Patient Taking Kratom and Multiple Serotonergic Agents. Journal of Pharmacy Practice. 36(6): 1523-1527.

Fernandes CT, Iqbal U, Tighe SP, Ahmed A. 2019. Kratom-Induced Cholestatic Liver Injury and Its Conservative Management. Journal of Investigative Medicine High Impact Case Reports. 7: 1-4.

Griffiths CL, Gandhi N, Olin JL. 2018. Possible kratom-induced hepatomegaly: A case report. Journal of the American Pharmacists Association. 58(5): 561-563.

Haider M, Shah N, Yazdani A. 2023. Kratom-induced common bile duct dilation. Baylor University Medical Center Proceedings. 36(1): 116-117.

Hall A, Hall D. 2021. Kratom Ingestion and Emergency Care: Summary and a Case Report. Journal of Emergency Nursing. 47(4): 551-556.

Khan MZ, Saleh MA, Alkhayyat M, Roberts DE, Lindenmeyer CC. 2021. Multiorgan Dysfunction Related to Kratom Ingestion. ACG Case Reports Journal. 8(8): 1-3.

LeSaint KT, Yin S, Sharma A, Avery BA, McCurdy CR, Waksman JC. 2022. Acute Renal Insufficiency Associated With Consumption of Hydrocodone- and Morphine-Adulterated Kratom (Mitragyna Speciosa). Journal of Emergency Medicine. 63(1): e28-e30.

Martin G, Collins DP, Valenzuela H. 2022. Life-Threatening Hyponatremia Secondary to Chronic Kratom Use: A Case Presentation. Cureus. 14(9): 1-4.

Matos-Casano HA, Nanduri S. 2021. Transient Paralysis: A Novel Expression of Kratom Toxicity in Humans. Neurology: Clinical Practice. 11(1): e28-e29.

Mousa MS, Sephien A, Gutierrez J, O'Leary C. 2018. N-Acetylcysteine for Acute Hepatitis Induced by Kratom Herbal Tea. American Journal of Therapeutics. 25(5): e550-e551.

Nayak LJ, Sondhi AR, Westerhoff M. 2021. History! Gastroenterology. 160(6): e5-e6.

Osborne CS, Overstreet AN, Rockey DC, Schreiner AD. 2019. Drug-Induced Liver Injury Caused by Kratom Use as an Alternative Pain Treatment Amid an Ongoing Opioid Epidemic. Journal of Investigative Medicine High Impact Case Reports. 7: 1-5.

Peran D, Stern M, Cernohorsky P, Sykora R, Popela S, Duska F. 2023. Mitragyna speciosa (Kratom) poisoning: Findings from ten cases. Toxicon. 225: 1-5.

Riverso M, Chang M, Soldevila-Pico C, Lai J, Liu X. 2018. Histologic Characterization of Kratom Use-Associated Liver Injury. Gastroenterology Research. 11(1): 79-82.

Roma K, Mohammed S, Sieck B, Naik K, Wahid S. 2023. Kratom-induced acute liver injury: A case study and the importance of herbal supplement regulation. Journal of Hepatology. 79(2): 581-584.

Sangani V, Sunnoqrot N, Gargis K, Ranabhotu A, Mubasher A, Pokal M. 2021. Unusual Presentation of Kratom Overdose With Rhabdomyolysis, Transient Hearing Loss, and Heart Failure. Journal of Investigative Medicine High Impact Case Reports. 9: 1-4.

Sheikh M, Ahmed N, Gandhi H, Chen O. 2021. Report of ventricular fibrillation in a 44-year-old man using kratom. BMJ Case Reports. 14: 1-3.

Singh V, Mulla N, Wilson JL, Umansky A, Lee J, Stead T, et al. 2020. Intractable nausea and vomiting in naive ingestion of kratom for analgesia. International Journal of Emergency Medicine. 13: 1-4.

Tatum WO, Hasan TF, Coonan EE, Smelick CP. 2018. Recurrent seizures from chronic kratom use, an atypical herbal opioid. Epilepsy & Behavior Case Reports. 10: 18-20.

Torres-Ortiz A, Al Zein S, Alqudsi M. 2022. A Case of Hyperkalemia Induced by Kratom (Mitragyna speciosa). Cureus. 14(4): 1-5.

Vanani NB, Stevanovic SG, Stevanovic N. 2023. Adverse Drug Interaction Between Kratom and Amitriptyline With Gastrointestinal and Mild Hepatic Effects. Cureus. 15(1): 1-4.

Zuberi M, Guru PK, Bansal V, Diaz-Gomez J, Grieninger B, Alejos D. 2019. Undifferentiated Shock and Extreme Elevation of Procalcitonin Related to Kratom Use. Indian Journal of Critical Care Medicine. 23(5): 239-241.
